# Deep learning sheds new light on non-orthogonal optical multiplexing

**DOI:** 10.1038/s41377-024-01460-y

**Published:** 2024-05-06

**Authors:** Zhengzhong Huang, Liangcai Cao

**Affiliations:** Department of Precision Instruments, Tsinghua University, Beijing, China

**Keywords:** Fibre optics and optical communications, Imaging and sensing

## Abstract

A deep neural network for non-orthogonal input channel encoding is proposed to recover speckle images through a multimode fiber. This novel approach could shed new light on the non-orthogonal optical multiplexing over a scattering medium.

Optical multiplexing is one of the key technologies of modern fiber communication, where wavelength division multiplexing, polarization division multiplexing, and space division multiplexing are typical examples^1–5^. The fundamental enabler of division multiplexing is orthogonality among multiplexing channels in a single-mode fiber, where the cost of digital signal postprocessing can be substantially reduced^1,3^. Distinct from the single-mode fiber, a multimode fiber that supports thousands of propagation modes could be a potential candidate for achieving high-throughput transmission of optical information^5^.

Nowadays, deep learning has become a popular and promising tool in many aspects of optics, including but not limited to holography^6,7^, optical metrology^8^, inverse design of optical devices^9^, and computational imaging^10–13^, etc. Particularly, deep neural networks have been applied in tackling multiple scattering problems in strongly scattering media^14–18^, where the disturbed information encoded on the input wavefront can be effectively retrieved from a speckle output in a one-to-one manner.

In a recent publication by Pan et al. in *Nature Communications*, the research team introduced a concept of non-orthogonal optical multiplexing through a multimode fiber by the deep neural network^19^, where multiple information sources encoded in spatial overlapping non-orthogonal input channels mediated by the same polarization and wavelength can be demultiplexed using a single-shot intensity detection. In this process, the information encoded in phase and amplitude dimensions is thoroughly scrambled, resulting in a chaotic speckle output.

This important work solved an inversion problem that the complicated mapping relationship between two non-orthogonal multiplexing input channels and a speckle output of the multimode fiber can be acquired using a deep neural network (Fig. 1), where the neural network consists of a fully connected network and a ConvNet. The multiplexed information at the input of the multimode fiber dramatically increases the complexity of information retrieval from a single speckle image compared with the one-to-one mapping case. Essentially, this one-to-multiple mapping is enabled by the multiple scattering of the multimode fiber that slightly different inputs will result in distinct speckle patterns at the output of the multimode fiber, facilitating the distinct identification of given non-orthogonal inputs in parametric space. Even multiplexing uncorrelated binary information and natural scene images with diverse properties can be effectively retrieved with high fidelity utilizing a single-shot speckle pattern, indicating the possibility of generalizing the proposed network to other scenarios of scattering.

**Fig. 1:**
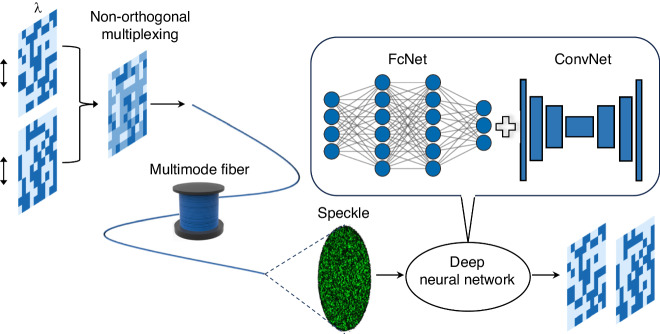
Schematic of non-orthogonal optical multiplexing over an multimode fiber. Non-orthogonal optical multiplexing via spatial overlapped input channels with the same polarization and wavelength through a multimode fiber using deep learning

Utilizing advanced neural networks can further boost the fidelity of non-orthogonal multiplexing over a strongly scattering medium. As envisioned by the authors, one of the future works that can be carried out is the combination of a neural network with a physical model to release the data burden and facilitate the broader applications of this concept in other disciplines.
